# Detection of virus-specific intrathecally synthesised immunoglobulin G with a fully automated enzyme immunoassay system

**DOI:** 10.1186/1471-2377-7-12

**Published:** 2007-05-29

**Authors:** Jörg Schubert, Benedikt Weissbrich

**Affiliations:** 1 Institute of Virology and Immunology, University of Würzburg, Versbacher Str. 7, 97078 Würzburg, Germany

## Abstract

**Background:**

The determination of virus-specific immunoglobulin G (IgG) antibodies in cerebrospinal fluid (CSF) is useful for the diagnosis of virus associated diseases of the central nervous system (CNS) and for the detection of a polyspecific intrathecal immune response in patients with multiple sclerosis. Quantification of virus-specific IgG in the CSF is frequently performed by calculation of a virus-specific antibody index (AI). Determination of the AI is a demanding and labour-intensive technique and therefore automation is desirable. We evaluated the precision and the diagnostic value of a fully automated enzyme immunoassay for the detection of virus-specific IgG in serum and CSF using the analyser BEP2000 (Dade Behring).

**Methods:**

The AI for measles, rubella, varicella-zoster, and herpes simplex virus IgG was determined from pairs of serum and CSF samples of patients with viral CNS infections, multiple sclerosis and of control patients. CSF and serum samples were tested simultaneously with reference to a standard curve. Starting dilutions were 1:6 and 1:36 for CSF and 1:1386 and 1:8316 for serum samples.

**Results:**

The interassay coefficient of variation was below 10% for all parameters tested. There was good agreement between AIs obtained with the BEP2000 and AIs derived from the semi-automated reference method.

**Conclusion:**

Determination of virus-specific IgG in serum-CSF-pairs for calculation of AI has been successfully automated on the BEP2000. Current limitations of the assay layout imposed by the analyser software should be solved in future versions to offer more convenience in comparison to manual or semi-automated methods.

## Background

The determination of virus-specific immunoglobulin G (IgG) antibodies in cerebrospinal fluid (CSF) is an important tool for the diagnosis of virus-associated diseases of the central nervous system (CNS) and for the detection of a polyspecific intrathecal immune response in patients with multiple sclerosis (MS) [1,2]. Quantification of virus-specific IgG in the CSF is frequently performed by calculation of a virus-specific antibody index (AI) [3]. The AI is the ratio of the CSF/serum quotient of virus-specific IgG (Q_spec_) and of the CSF/serum quotient of total IgG (Q_IgG_), i. e. AI = Q_spec_/Q_IgG_. The replacement of Q_IgG _by Q_lim _has been proposed as a correction in cases of an intrathecal IgG synthesis [3]. Q_lim _represents the upper limit of the Q_IgG _under the assumption that the IgG fraction in the CSF originates only from blood. Q_lim _can be calculated for an individual patient from the CSF/serum quotient of albumin (Q_Alb_) [4].

The determination of virus-specific antibodies is usually performed using enzyme immunoassays. In order to achieve a high precision, it is advisable to analyse CSF and serum simultaneously with reference to a standard curve [3]. Because the IgG content of CSF samples is usually low, modifications of standard serum enzyme immunoassays are necessary to increase the sensitivity of the detection of virus-specific antibodies. Possible modifications include increased incubation times and conjugate concentrations [3,5]. With respect to the working dilutions of serum and CSF, several aspects have to be considered. Highly concentrated CSF samples may lead to unspecific matrix effects. On the other hand, dilution of CSF samples will decrease the sensitivity of antibody detection. The ratio of the serum and CSF working dilutions should resemble the concentration gradient of IgG between serum and CSF, which is approximately 200:1 for healthy adults [3].

Overall, AI determination is a demanding and labour-intensive technique and automation is desirable. Therefore, we evaluated the precision and the diagnostic value of a fully automated enzyme immunoassay for the detection of virus-specific IgG in serum and CSF using the analyser BEP2000 (Dade Behring).

## Methods

### Samples

The serum and CSF samples used in this study had been sent to the virology laboratory at the University of Würzburg for routine testing of intrathecal synthesis for measles, rubella, *varicella-zoster* (VZV), and herpes simplex virus (HSV) IgG. Samples of the following groups were used in this study: psychiatric patients with normal CSF findings (n = 29) who were tested for exclusion of inflammatory CNS disease; patients with a diagnosis of subacute sclerosing panencephalitis (SSPE; n = 9), VZV meningitis or encephalitis (n = 12), HSV encephalitis (n = 10), and MS (n = 22).

The requested AI determination was performed routinely in a semi-automated fashion after arrival of the samples in the virology laboratory. Remaining material was stored at -20°C for a mean period of 3 years (range 0 – 10 years). For evaluation of the novel fully automated AI determination method, the stored aliquots were tested and the AI values of the routine determinations were compared with results obtained during the evaluation study. The study was carried out in compliance with the Helsinki declaration and was approved by the ethics committee of the medical faculty at the University of Würzburg.

### Semi-automated antibody index determination with the BEP III

**Figure 1:**
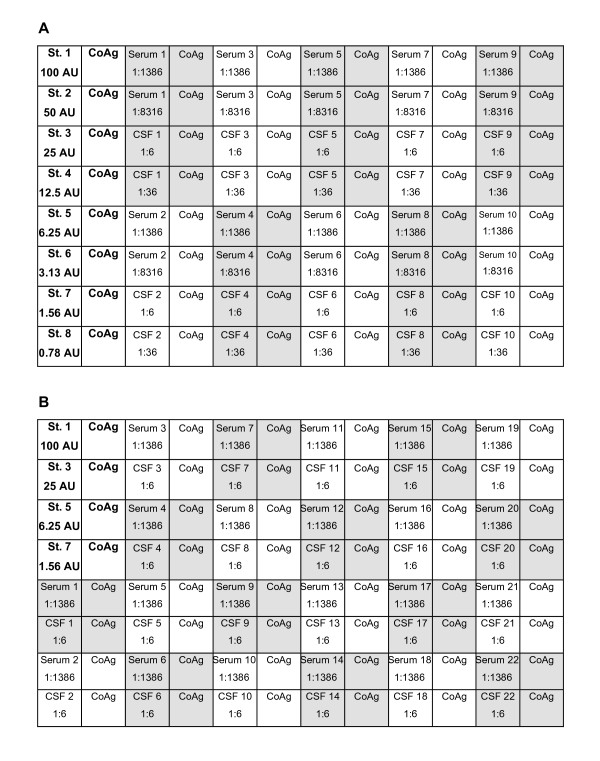
**Pipetting schemes of the AI determination methods**. AI determinations were performed with two microtiter plate layouts. Every second strip of the microtiter plates is coated with control antigen (CoAg). Standard positions (St.) with the corresponding arbitrary units (AU) are presented in bold type. Shading indicates pairs of serum and CSF samples. (A) 8-point-standard curve and two dilutions each for serum and CSF. (B) 4-point-standard-curve and one dilution each for serum and CSF.

Antibody index determination for IgG antibodies against measles, rubella, VZV, and HSV was originally performed with a semi-automated method as described previously [6]. Enzyme immunoassay reagents contained in Dade Behring Enzygnost IgG test kits (Dade Behring, Marburg, Germany) were used for this purpose. All dilution and pipetting steps of the standards and samples were performed manually. The microtiter plates (MTP) were processed using a Behring Elisa Processor III (Dade Behring). One of two pipetting schemes was used for the routine AI determination (Figure 1). Either an 8-point-standard curve with 1:2 dilution steps, or a 4-point-standard curve with 1:4 dilution steps was pipetted on the first strip of each microtiter plate. Commercial plasma pools such as Standard Human Plasma (SHP, Dade Behring), which serve as reference material in clinical chemistry, were used for the standard dilutions. The starting dilution of each standard curve, which varied according to the content of virus-specific IgG, was assigned a value of 100 arbitrary units (AU). Originally, serum and CSF samples were tested in two dilutions each, namely 1:1386 and 1:8316 for serum, and 1:6 and 1:36 for CSF, respectively (Figure 1a). Later, serum and CSF were tested only in one dilution each (Figure 1b). Where appropriate, higher dilutions in steps of 1:6 were performed. In order to increase the sensitivity of the IgG detection of the enzyme immunoassay compared to the intended use of the Enzygnost test kits, some modifications of the assay conditions were made. These included a serum incubation time of 180 min instead of 60 min, a conjugate dilution of 1:31 instead of 1:51, and a conjugate incubation time of 90 min instead of 60 min. The washing, substrate and stop solution steps and the photometer reading were performed according to the instructions of the Enzygnost test kits. AUs of serum and CSF samples were calculated with reference to the standard curve included on each MTP. Subsequently, the Q_spec _was calculated according to the formula Qspec=AUCSF*dilutionCSFAUserum*dilutionserum
 MathType@MTEF@5@5@+=feaafiart1ev1aaatCvAUfKttLearuWrP9MDH5MBPbIqV92AaeXatLxBI9gBaebbnrfifHhDYfgasaacH8akY=wiFfYdH8Gipec8Eeeu0xXdbba9frFj0=OqFfea0dXdd9vqai=hGuQ8kuc9pgc9s8qqaq=dirpe0xb9q8qiLsFr0=vr0=vr0dc8meaabaqaciaacaGaaeqabaqabeGadaaakeaacqqGrbqudaWgaaWcbaGaee4CamNaeeiCaaNaeeyzauMaee4yamgabeaakiabg2da9maalaaabaGaeeyqaeKaeeyvau1aaSbaaSqaaiabboeadjabbofatjabbAeagbqabaGccqqGQaGkcqqGKbazcqqGPbqAcqqGSbaBcqqG1bqDcqqG0baDcqqGPbqAcqqGVbWBcqqGUbGBdaWgaaWcbaGaee4qamKaee4uamLaeeOrayeabeaaaOqaaiabbgeabjabbwfavnaaBaaaleaacqqGZbWCcqqGLbqzcqqGYbGCcqqG1bqDcqqGTbqBaeqaaOGaeeOkaOIaeeizaqMaeeyAaKMaeeiBaWMaeeyDauNaeeiDaqNaeeyAaKMaee4Ba8MaeeOBa42aaSbaaSqaaiabbohaZjabbwgaLjabbkhaYjabbwha1jabb2gaTbqabaaaaaaa@662B@. The AI was determined as Q_spec_/Q_IgG _when Q_IgG _< Q_lim _or as Q_spec_/Q_lim _when Q_IgG _> Q_lim _as suggested by Reiber et al. [3]. Values of AI > 1.4 are indicative of an intrathecal synthesis of virus-specific IgG.

### Automated antibody index determination with the BEP2000

The fully automated AI determination was performed with the BEP2000 (Dade Behring), a walk-away enzyme immunoassay analyser with a pipetting device for disposable tips. In contrast to the BEP III, the BEP2000 allows automation of the dilution and pipetting steps of the standard curves and of the serum and CSF samples. The pipetting scheme and the enzyme immunoassay parameters were similar to the semi-automated method with some modifications as described below. Reagents of the Dade Behring Enzygnost IgG test kits were used for the enzyme immunoassays and Standard Human Plasma (Dade Behring) was used as standard material. The initial evaluations were performed with an 8-point-standard curve and two dilutions each of serum and CSF (Figure 1a). In order to optimise the intraassay variation and to obtain similar absolute optical density values with the BEP2000 compared to the semi-automated method with the BEP III, the conjugate incubation time was reduced from 90 min to 60 min and the dispensed volume of the substrate and stopping solution was reduced from 100 μl to 75 μl.

After successful implementation of the 8-point-standard curve method on the BEP2000, the method was adapted to the 4-point-standard curve method with one dilution each for serum and CSF (Figure 1b) in order to minimise reagent costs. For both the 8-point- and 4-point-standard curve method, the dilutions of serum and CSF samples are fixed, which meant that CSF and serum samples were prediluted manually where necessary.

### Calculation of intraassay and interassay variation

To determine the intraassay and interassay variation of the automated AI determination method, an artificial serum-CSF-pair was used as run control. It was produced from a plasma pool containing IgG against measles virus, rubella virus, VZV and HSV. Undiluted plasma pool was aliquoted and stored at -20°C to serve as "serum run control" (SRC). For the artificial CSF sample, the plasma pool was manually prediluted 1:231 in sample buffer, aliquoted and also stored at -20°C (CSF run control, CRC). During the AI determination, the CRC is treated as CSF sample and therefore diluted 1:6. Taking into consideration the manual predilution of 1:231, the final dilution with respect to the undiluted plasma pool is 1:1386. This corresponds to the test dilution of serum samples in the AI enzyme immunoassay. Thus, the optical density and the AU values of SRC and CRC should theoretically be equal. The ratio of AU_CRC_/AU_SRC _can serve as a quality control parameter for the AI determination method and is used for calculation of intraassay and interassay coefficients of variation. In order to validate the use of the pre-diluted serum pool as a CSF surrogate marker, we compared intraassay variation of a serum-CSF-pair that was available in large enough quantity with the artificial serum-CSF-pair. The results of the coefficients of variation of both sample pairs were in a similar range.

## Results

**Figure 2:**
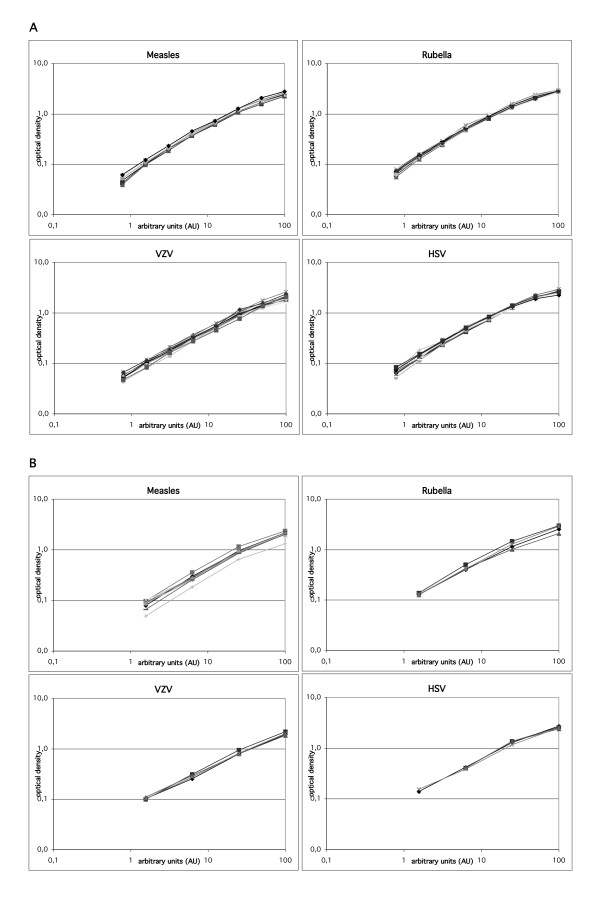
**Standard curves obtained with the BEP2000**. Log-log diagrams of representative 8-point-standard curves (A) and 4-point-standard curves (B) obtained with the BEP2000 for measles, rubella, VZV, and HSV. Each curve represents a separate run. Arbitrary units assigned to the standard dilutions are shown on the x-axis. Optical density values (OD) are shown on the y-axis.

In order to obtain an impression of the performance of the automated AI determination on the BEP2000, the dilution linearity of the standard curves and the intraassay and interassay coefficients of variation were tested in initial experiments. Representative examples of 8-point-dilutions standard curves are shown in Figure 2a. The dilution linearity was good and the linear range of the curves extended from 0.100 to at least 2.500 optical density.

To determine the imprecision of the method, the coefficient of variation of the ratio AU_CRC_/AU_SRC _was calculated for each virus by testing a run control, which represented an artificial serum-CSF-pair and was available in large quantity to allow repeated testing (Table 1). The interassay coefficient of variation was around 5 – 9% for all parameters tested.

**Table 1:** Intraassay and interassay variation of the ratio AU_CRC_**/AU**_SRC _^1^

	semi-automated method				8-point-standard curve BEP2000				4-point-standard curve BEP2000			
	Measles	Rubella	VZV	HSV	Measles	Rubella	VZV	HSV	Measles	Rubella	VZV	HSV
**Intraassay**												
n					10	10	10	10	10	10	10	10
Mean	n. d.^2^	n. d.	n. d.	n. d.	0.95	0.92	0.95	0.91	0.89	0.89	0.91	0.91
Standard deviation	n. d.	n. d.	n. d.	n. d.	0.04	0.06	0.04	0.05	0.03	0.01	0.06	0.03
Coefficient of variation	n. d.	n. d.	n. d.	n. d.	4.5%	6.9%	4.5%	5.1%	3.2%	1.6%	6.8%	3.1%
												
**Interassay**												
n	20	19	22	24	10	10	11	11	20	19	23	19
Mean	1.10	1.06	1.04	1.09	0.99	0.97	0.96	0.96	1.05	1.07	1.14	1.06
Standard deviation	0.15	0.11	0.09	0.13	0.05	0.06	0.08	0.07	0.10	0.04	0.07	0.08
Coefficient of variation	14.0%	10.3%	8.9%	12.2%	5.3%	6.2%	8.6%	6.9%	9.6%	4.1%	6.3%	7.3%

^1^CRC and SRC represent an artificial serum-CSF-pair available in sufficient quantity to be repeatedly tested. The theoretical value of AU_CRC_/AU_SRC _is 1. For further details, see method.

^2 ^n. d. – not determined

To evaluate the clinical usefulness of the fully automated method, archived serum-CSF-pairs that had originally been tested for AI values in a semi-automated fashion were retested with the BEP2000. The results of normal controls are shown in Table 2. Results obtained with the BEP2000 and AI values derived from the semi-automated reference method were in good agreement. The standard deviations of the BEP2000 results were lower than the standard deviations of the semi-automated method. AI results of the different samples groups (MS, VZV and HSV infection, SSPE, normal controls) were highly correlated (Table 3). Detailed results of the MS group are presented in Table 4. Overall, there was good qualitative (cut-off 1.4) and quantitative agreement between both methods. For eight of the 88 AI values obtained with each method, there were qualitative differences between the semi-automated method and the 8-point-standard curve method on the BEP2000 (underlined in Table 4). However, the discrepant values varied only in a narrow range, and none of the samples was found as falsely normal with either method when all four viruses tested were considered.

**Table 2:** Antibody index results of normal controls

	semi-automated method				8-point-standard curve BEP2000				4-point-standard curve BEP2000			
	Measles	Rubella	VZV	HSV	Measles	Rubella	VZV	HSV	Measles	Rubella	VZV	HSV
Mean	0.81	0.93	0.96	1.06	0.89	0.91	0.96	1.03	0.83	0.91	0.94	1.02
Standard deviation	0.17	0.12	0.18	0.20	0.13	0.11	0.13	0.14	0.15	0.13	0.15	0.18
Minimum	0.50	0.70	0.70	0.80	0.67	0.68	0.72	0.73	0.57	0.70	0.75	0.71
Maximum	1.30	1.10	1.30	1.30	1.16	1.10	1.18	1.30	1.13	1.17	1.42	1.34

**Table 3:** Correlation coefficients r for pairwise comparisons of the AI values obtained with different methods for patient samples (MS, HSV and VZV infection, SSPE) and normal controls

Method comparison	Measles	Rubella	VZV	HSV
semi-automated and BEP2000 (8-point-standard curve)	0.95	0.98	0.97	0.98
semi-automated and BEP2000 (4-point-standard curve)	0.94	0.99	0.97	0.98
BEP2000 (8-point and 4-point-standard curve)	0.99	0.99	0.96	0.99

**Table 4:** Antibody index results of MS patients. AI > 1.4 is indicative of intrathecal synthesis of virus-specific IgG. Qualitative differences between the semi-automated method and the 8-point-standard curve method on the BEP2000 are underlined. Qualitative differences between the 4-point-standard curve method (BEP2000) and the other two methods are shown in bold face and italics

		semi-automated method				8-point-standard curve BEP2000				4-point-standard curve BEP2000			
sample	IgG index^1^	Measles	Rubella	VZV	HSV	Measles	Rubella	VZV	HSV	Measles	Rubella	VZV	HSV
1	1.37	2.7	neg.^2^	9.5	neg.	3.7	neg.	11.0	neg.	4.0	neg.	11.4	neg.
2	1.31	1.7	1.6	17.5	0.4	1.6	1.8	15.2	0.6	***1.4***	1.9	20.3	1.2
3	1.08	3.1	0.7	1.6	0.5	2.9	0.9	1.4	0.7	1.9	0.8	1.2	0.7
4	0.87	5.9	0.7	11.9	1.9	7.7	0.8	10.5	2.0	***1.3***	0.7	10.3	***1.1***
5	1.18	42.2	6.9	1.8	3.0	24.7	9.7	3.1	2.4	22.1	11.6	2.7	2.2
6	1.36	5.9	14.3	9.4	neg.	6.1	14.5	9.3	neg.	4.9	12.6	8.8	neg.
7	1.88	43.4	0.8	2.2	neg.	35.8	1.6	3.5	neg.	29.4	1.3	2.0	neg.
8	1.15	2.6	2.9	1.0	0.5	3.2	2.5	1.0	0.7	4.1	2.6	0.8	0.5
9	1.08	2.8	1.2	0.6	0.6	5.9	3.9	1.7	0.7	n. d. ^3^	n. d.	n. d.	n. d.
10	0.73	2.5	1.6	1.5	2.1	2.0	1.4	0.8	1.2	2.6	1.2	0.8	0.9
11	3.19	11.8	3.7	1.6	neg.	5.0	3.1	1.6	neg.	3.7	2.6	***1.2***	neg.
12	0.99	6.3	3.4	7.6	0.7	9.0	2.8	2.9	0.6	n. d.	n. d.	n. d.	n. d.
13	0.98	1.1	2.2	2.3	1.2	1.1	5.8	1.4	0.9	n. d.	n. d.	n. d.	n. d.
14	1.78	1.3	9.5	1.9	neg.	1.2	12.0	3.3	neg.	1.4	10.4	3.7	neg.
15	2.20	4.4	1.8	4.1	neg.	3.5	1.8	3.4	neg.	3.5	1.6	2.9	neg.
16	2.27	3.0	2.6	n. a.^4^	2.1	4.5	4.2	3.4	3.3	3.2	3.2	3.0	1.6
17	1.21	7.0	4.5	0.9	0.4	5.3	4.0	1.0	0.7	8.4	5.8	0.9	0.6
18	4.23	10.1	44.7	10.7	1.0	8.8	37.4	10.4	0.9	9.1	41.7	10.5	1.0
19	2.78	12.3	11.0	17.5	0.9	7.1	11.4	19.1	1.0	7.7	13.6	19.3	1.0
20	2.04	5.7	3.8	1.9	2.6	8.6	5.8	2.2	1.6	8.9	5.2	2.1	1.9
21	2.65	2.1	4.2	3.4	7.1	2.3	3.0	5.3	5.8	1.9	1.8	3.6	5.7
22	0.98	11.4	1.5	2.5	1.2	5.3	1.9	2.4	1.2	6.4	1.9	2.3	1.0

^1 ^IgG index = Q_IgG_/Q_Alb_

^2 ^neg. – virus specific IgG below the detection limit; an AI could not be calculated

^3 ^n. d. – not determined because of insufficient sample volume

^4 ^n. a. – a VZV AI value from routine determinations with the semi-automated method was not available

Because of the good precision and reproducibility of the results obtained with the 8-point-standard curve pipetting scheme (Figure 1a), the automated method was modified in order to minimise reagent costs. The standard curve was reduced from 8 points to 4 points, and serum and CSF were tested in only one dilution each (Figure 1b). Representative standard curves and coefficients of variation obtained with the modified method are shown in Figure 2b and Table 1. The results were comparable to the 8-point-standard curve pipetting scheme. Finally, clinical samples were also tested with the 4-point-standard curve pipetting scheme. For the normal controls, there was a good agreement between the results obtained with the semi-automated method, the 8-point and the 4-point-standard curve method (Table 2). AI results of the different samples groups were highly correlated between the semi-automated method and the 4-point-standard curve method on the BEP2000 as well as between the 4-point- and 8-point-standard curve method on the BEP2000 (Table 3). For four of 76 AI values obtained with the 4-point-standard curve method in the MS sample group, there were qualitative discrepancies compared to the other two methods. However, at least one AI was elevated in all of the MS samples and none of them was found to be entirely normal (Table 4).

## Discussion

Detection of intrathecal synthesis of virus-specific antibodies is an important tool in the diagnosis of inflammatory diseases of the CNS. Various methods have been proposed for this purpose [2,6,7]. The required specifications of the methods are partially dependent on their clinical use. Sensitive detection of a polyspecific antiviral immune response in patients with chronic inflammatory CNS disease, such as MS, can be achieved by AI determination and is especially demanding with respect to assay precision [1,8]. While automated assays have become an integral part of serological laboratories in recent years, antibody index determination in serum-CSF-pairs has been difficult to automate so far because of its complexity. In this study, we have successfully advanced an established semi-automated method for AI determination to full automation on the enzyme immunoassay analyser BEP2000.

In order to obtain a high precision of the antibody index values, the optical densities of serum and CSF in the enzyme immunoassay should fall in a similar range. Therefore, working dilutions of serum and CSF were chosen as to resemble the concentration gradient of IgG between serum and CSF, which resulted in rather high serum dilutions. As a consequence, six aspiration and three dispensing steps were necessary to obtain the serum working dilution 1:1386, and two pipetting steps were required for the CSF dilution (1:6). Because the inaccuracies of each pipetting step add up to the sum error of Q_spec_, a high precision of the pipetting process is required. This aim has been achieved by the automated method as demonstrated by the low coefficient of variation of the run controls. In our experience, the precision of the BEP2000 tends to be higher than the precision that can be achieved by manual pipetting in a routine diagnostic setting with rotating personnel and time constraints.

This observation is also supported by the correlation coefficients for the measles virus AI values (Table 3). Correlation between the values obtained with the automated 4-point- and 8-point-standard curve methods was higher than the correlation between either automated method and the semi-automated method. This is probably attributed to the inclusion of samples from SSPE patients in the study. These samples are characterised by extremely high titres of measles virus IgG. As a result, serum and CSF samples have to be tested in very high dilutions in order to obtain optical density values in the linear range of the enzyme immunoassay. Common working dilutions for these samples are 1:299376 for serum (i. e. 8316 × 6 × 6) and 1:7776 for CSF (i. e. 36 × 6 × 6 × 6). Due to these additional dilution steps, the imprecision of the measles virus AI determinations of the SSPE samples derived from routine testing with the semi-automated method is probably higher than the imprecision of the automated determinations performed within this study.

An additional explanation for discrepancies between AI values obtained with different methods, apart from imprecision of pipetting and enzyme immunoassay processing, may be lot-to-lot variation of the antigen composition of the MTP. In fact, we have observed some qualitative AI discrepancies when MS samples were tested in parallel with five different lots of measles MTP (data not shown). In contrast to a polyclonal immune response directed against a broad spectrum of epitopes, effects of antigen variation may become apparent more easily in cases of an oligoclonal immune response, which is directed against only a few antigenic sites.

The 8-point-standard curve assay layout with two dilutions for each serum and each CSF sample, which was originally employed, was useful for evaluation purposes but is very reagent consuming. The assay was made more cost-effective by using only one instead of two sample dilutions and by using a 4-point standard curve instead of an 8-point standard curve. While only 10 serum-CSF-pairs can be tested on one MTP with the 8-point-standard curve layout (the Enzygnost assays include control antigen strips), 22 pairs can be tested with the 4-point-standard curve and the single dilution layout. Obviously, the reductions of sample dilutions will lead to a higher retest rate because optical density values of the starting dilutions may fall in the saturation range of the standard curve. Advantages and disadvantages of the different layouts have to be balanced by each laboratory depending on the individual needs.

A limitation of both the semi-automated and the automated method are the long serum and conjugate incubation times. The extension of these incubation times compared to the standard IgG assay was originally implemented in analogy to the method described by Reiber and Lange [3]. The duration of processing one plate on the BEP2000 from start to finish is approximately 6 hours. Because the BEP2000 has only four MTP incubators, it can handle only four plates at a time. Thus, the throughput is 40 results per working day with the 8-point standard curve method and 88 results per working day with the 4-point standard curve method. Since the BEP2000 is a walk-away analyser, it may be possible to double the throughput by reloading the machine at the end of the working day. Obviously, shortening the incubation times would further increase the throughput. Assay modifications to this end are currently ongoing.

Because the CSF volume available for serology is often limited, minimising the required sample volume is important. In the assays described in this paper, 160 μl of CSF are necessary for determination of antibody indices for measles, rubella, VZV, and HSV. This volume includes 4 × 20 μl = 80 μl required for control antigen wells which are part of the Dade Behring test kits. However, in our experience reaction of CSF samples with control antigen in IgG enzyme immunoassays is extremely rare. Therefore, pipetting of the control antigen well has been omitted for CSF samples in a modified semi-automated version of the antibody index assay, which is currently under evaluation.

A shortcoming of the automated antibody index method is the limited flexibility of the current version of the analyser software. The dilution steps are invariable and attributed to fixed positions of the assay layout. Thus, testing of samples in higher dilutions, when the absolute antibody titre exceeds the linear range of the standard curve, requires manual predilution steps. Testing of samples in lower dilutions is not possible at all. Furthermore, testing of some samples in one dilution and others in two dilutions on the same plate is not possible without manual sample splitting and predilution. While the automated method is well suited for initial testing of large numbers of serum-CSF-pairs, its use is more laborious for the antibody-titre-adapted retesting of those samples, for which an antibody index could not be calculated in the initial testing.

The method described in this paper is an in-house method and has not been officially validated by the manufacturer. As a consequence, the reagents used for this assay have not been validated for this purpose either. Especially the quality of the MTPs, coated with viral antigen, of the anti-human IgG conjugate, and of the plasma pools used for the standard curves, will have a significant influence on the optical density values of the samples and the standard curves. Establishment of specifications for the use of these reagents in CSF diagnosis, which take lot-to-lot variations into account, is therefore recommended. It is particularly important to define upper and lower limits of the linear range of the standard curve, in order to ensure reporting of correct antibody index results.

## Conclusion

Determination of virus-specific IgG has been successfully automated on the BEP2000. The interassay variation of the AIs obtained with the BEP2000 was low and there was a good agreement between the automated and the semi-automated method. Automation may ultimately lead to a better standardisation of CSF analysis. Necessary improvements of the analyser software will hopefully increase the flexibility of the automated method in the future.

## Abbreviations

AI, antibody index; AU, arbitrary units; BEP, Behring Elisa Processor; CNS, central nervous system; CRC, CSF run control; CSF, cerebrospinal fluid; HSV, Herpes simplex virus; MS, multiple sclerosis; MTP, microtiter plate; SSPE, subacute sclerosing panencephalitis; SRC, serum run control; VZV, varicella-zoster-virus.

## Competing interests

BW has received research and travel grants from Dade Behring.

## Authors' contributions

Both authors were responsible for establishing and optimization of the AI determinations on the BEP2000, for data analysis, and for writing the manuscript.

## Pre-publication history

The pre-publication history for this paper can be accessed here:


